# Measuring the Density of States of the Inner and Outer Wall of Double-Walled Carbon Nanotubes

**DOI:** 10.3390/nano8060448

**Published:** 2018-06-19

**Authors:** Benjamin A. Chambers, Cameron J. Shearer, LePing Yu, Christopher T. Gibson, Gunther G. Andersson

**Affiliations:** 1 Flinders Centre for NanoScale Science and Technology, Flinders University, Adelaide SA 5001, Australia; benjamin.chambers@flinders.edu.au (B.A.C.); cameron.shearer@adelaide.edu.au (C.J.S.); leping.yu@flinders.edu.au (L.Y.); 2 Department of Chemistry, The University of Adelaide, Adelaide SA 5005, Australia

**Keywords:** single-walled and double-walled carbon nanotubes, density of states, electron spectroscopy

## Abstract

The combination of ultraviolet photoelectron spectroscopy and metastable helium induced electron spectroscopy is used to determine the density of states of the inner and outer coaxial carbon nanotubes. Ultraviolet photoelectron spectroscopy typically measures the density of states across the entire carbon nanotube, while metastable helium induced electron spectroscopy measures the density of states of the outermost layer alone. The use of double-walled carbon nanotubes in electronic devices allows for the outer wall to be functionalised whilst the inner wall remains defect free and the density of states is kept intact for electron transport. Separating the information of the inner and outer walls enables development of double-walled carbon nanotubes to be independent, such that the charge transport of the inner wall is maintained and confirmed whilst the outer wall is modified for functional purposes.

## 1. Introduction

Carbon nanotubes (CNTs) were observed as early as 1972 [1]; however, the first report of them was made by Iijima in 1991 [2]. CNTs consist of a varying number of coaxial tubes of graphite sheets from a single tube, known as a single-walled carbon nanotube (SWCNT), to many coaxial tubes classified as multiwalled carbon nanotubes (MWCNTs). CNTs are known for their electronic properties due to its sp^2^ bonding network, ranging from semiconducting to metallic behaviour with the possibility of electron transport without backscattering [3]. At present, three main methods are used to fabricate CNTs; chemical vapour deposition [4,5,6], arc discharge [1,7], and laser ablation [8]. The electronic structure of carbon nanotubes depends strongly on the production and purification methods as any surface modification, defects, or contamination will disrupt the sp^2^ network creating areas of sp^3^ hybridisation resulting in the degradation of the favourable electronic properties. Furthermore, the use of CNTs within an electronic device, or for application at an interface, typically requires functionalisation so they can be attached or fixed within a device. It has been shown that double-walled carbon nanotubes (DWCNTs) can be functionalised such that the outer wall of a DWCNT is defected; however, the inner wall is suggested to be protected sacrificially by the outer wall [9,10,11,12,13]. If the outside of a DWCNT has been modified for attachment, the inner tube is responsible for the electron transport within a device. It is therefore of interest to develop experimental techniques that enable the measurement of the valence electrons for both the inner and the outer walls of the CNTs such that one can optimise interfaces within electronic devices, such as tuning band gaps in solar cell junctions, supercapacitors, nanotransistors, or other semiconductor devices [14,15,16,17].

In the present work we aim to determine the density of states (DoS) of the inner and the outer wall of DWCNTs by applying electron spectroscopy. First, SWCNTs are measured using ultraviolet photoelectron spectroscopy (UPS) and metastable helium induced electron spectroscopy (MIES) to provide a reference using both methods for the same surface, which allows for a direct comparison of the instrumental techniques. Second, the DWCNTs will be examined with the separate methods providing electronic states of the inner and outer walls using UPS and the outer wall using MIES. This allows for a comparison of the DoS to determine the inner wall DoS. Raman spectroscopy has been applied to provide information regarding defects correlating with the observations of features in the DoS.

MIES is a surface sensitive valence electron spectroscopy technique, making it ideal to examine exclusively the electronic structure of the outer surface of materials like carbon nanotubes without contributions of the substrate [18]; or in the case of a DWCNT, the inner tube. The nature of the MIES measurement is determined by the interaction between the metastable Helium (He*) atoms and the sample, and results in the ejection of electrons measured by the spectrometer. This physical nature of the interaction is responsible for the perfect surface sensitivity.

Thus far, UPS has been applied to determine the valence electron structure of carbon nanotubes [19,20,21]. UPS has a probing depth of a few nm as a consequence of the mean free path of the excited electrons [22]. Thus, the probing depth of UPS exceeds the thickness of the outer wall of a CNT. Consequently, in UPS the superposition of the DoS of the multiple walls and substrate are detected. Therefore, it is almost impossible to separate in an UP spectrum the DoS contribution of the individual coaxial tubes and any contribution of the substrate.

Using a combination of ultraviolet photoelectron spectroscopy and metastable helium induced electron spectroscopy, the density of states of the inner and outer coaxial carbon nanotubes can be separated. When fabricating DWCNTs for use in electronic devices, the inner and outer walls have different purposes; the inner wall is responsible for charge transport whilst the outer wall is responsible for attachment or other functional reasons. Separating the characterisation of these coaxial tubes unlocks further refinement of fabricating DWCNTs for specific designs.

## 2. Materials and Methods

The SWCNTs were purchased from Carbon Solutions (P2-SWCNTs); purified with low functionality (i.e., the CNTs have a low number of functional groups), carbonaceous purity greater than 90% [23] and metal content less than 8 wt % from thermal gravimetric analysis in air. The DWCNTs were purchased from Sigma Aldrich (755141) with less than 10% metal content. The DWCNTs were treated for approximately 16 h in 3M HNO_3_ to dissolve any metal catalyst and amorphous carbon to match the purity of the P2-SWCNTs. The CNTs were suspended in N,N-dimethylformamide (Sigma-Aldrich, Castle Hill, Australia) and then filtered through 0.02 µm alumina filter (Whatman Anodisc, Bio-Strategy, Shanghai, China), which had previously been coated with 1 nm of chromium followed by 20 nm of gold using a magnetron sputter coating instrument (Q300T D, Quorum Technologies, East Sussex, UK). This allowed measurements of the CNTs without charging and without having to transfer to a different substrate.

The purity of the samples was verified with X-ray photoelectron spectroscopy (XPS) and Raman spectroscopy. XPS showed a composition of 97% C and 3% O and no impurities. The C 1s spectra were characteristic for sp^2^ hybridised C and are shown in the supplementary section in Figure S3. The C 1s spectra were similar to those published for highly oriented pyrolytic graphite (HOPG) [24]. Further, the Raman spectra were typical for SWCNTs and DWCNTs. No Raman spectra, corresponding to amorphous carbon, was observed for any sample indicating that the purity of the samples was sufficient for the present work.

The investigation of the CNTs was conducted using UPS in an ultra-high vacuum (UHV) system built by SPECS (Berlin, Germany) with a base pressure of a few 10^−10^ mbar. A two-stage cold cathode gas discharge from MFS (Claustal-Zellerfeld, Germany) was used to generate UV light (He I line) and metastable helium used for MIES (see below). The emitted electrons were detected via a hemispherical Phoibos 100 energy analyser from SPECS (Berlin, Germany). UPS was conducted with a pass energy of 10 eV. A bias of −10 V was applied to the samples for the UPS measurements. The angle of UV radiation and the analyser were 54° with respect to the sample normal.

UPS irradiated the sample with UV photons from the He I line (hν = 21.2 eV). The photons led to excitation of the valence electrons through the photoelectric effect. The surface sensitivity was limited by the electron mean free path to the upper 2–3 nm of the sample [22]. The binding energy of the electrons in the sample can be determined using Equation (1). E_kinetic_ is corrected for the bias of −10 V.

E_binding_ = 21.2 eV − E_kinetic_, (1)

Instead of using electromagnetic radiation, one can use metastable helium atoms also produced by the cold cathode gas discharge used to create the UV light. When the metastable helium atom He* (1s2s) approaches the surface, two different pathways for de-excitation of the He* can occur [25]. The first mechanism is resonant ionisation (RI) followed by Auger neutralisation (AN). The second mechanism is Auger de-excitation (AD). The details of the MIES technique has been discussed in a previous paper [24,26].

Equation (1) holds also for spectra exclusively based on the AD mechanism but with an excitation energy of 19.8 eV instead of 21.2 eV. AD-based spectra were directly correlated to the DoS of the sample and can be plotted either against the kinetic energy or the binding energy of the emitted electrons. The measurements of the CNTs had sufficient AD contribution to allow for a comparison of the MIES and UPS on the binding energy scale [27]. MIES spectra taken at low temperatures ensure high AD-dominant spectra allowing for the direct comparison with UPS (see supplementary section, Figures S1 and S2). AN-dominated spectra have broader features than AD-dominated spectra [25,27].

Raman spectra were acquired using a WITec alpha 300R Raman microscope at an excitation laser wavelength of 532 nm with a ×40 objective (numerical aperture 0.60). Approximately 30 Raman spectra were recorded per sample at approximately 3 different areas. Each area was 100 × 100 µm in size and each region of the sample was separated by hundreds of microns. Typical integration times were 10–30 s for 2–3 accumulations per spectrum.

Each sample was heated in situ from room temperature to a high temperature of 733 K and held for approximately 10 min; this facilitates water desorption and the removal of adventitious carbon, which can result in large variations in the MIE and UP spectra [28]. A filament located under the sample holder was used for heating. The samples were each cooled using a gas line carrying nitrogen with a segment of a coil being submerged in liquid nitrogen. Temperatures of 113 and 173 K were achieved for the SWCNT and the DWCNT, respectively. Measurements of the surfaces were conducted with UPS and MIES before being measured with Raman spectroscopy.

## 3. Results

### 3.1. Raman Spectroscopy

Raman spectroscopy is extremely sensitive to repetitive network structure within CNTs. The sp^2^ network experiences planar vibrations resulting in Raman features around 1580 cm^−1^ annotated as G peaks [29]. Any defects or disorder in the structure will give rise to a D band approximately 1350 cm^−1^. A ratio of the intensity of the G and D peaks (G:D ratio) can be made to indicate, on average, the type of bonding the carbon atoms have within the repetitive structure and provide a value to quantify the order or disorder for different samples [30,31]. A radial breathing mode (RBM) band is also observed in Raman spectroscopy when looking at CNTs [32]. Due to its cylindrical nature, vibrations of the atoms on average will allow for contracted and expanded diameters giving a mechanism to estimate the diameter of the CNTs [33]. The Raman band just below 2700 cm^−1^ (~2680 cm^−1^) is an overtone of the D band and is commonly called the G’ band [34]. Like the RBM bands, the position of the G’ peak is related to the diameter of the nanotubes and the shape can also give an indication of whether a nanotube sample is single-walled or double-walled. For single-walled nanotubes the G’ band is typically a single symmetric peak, but for double-walled some broadening and asymmetry can potentially be observed. This is due to the inner and outer nanotubes in the double-walled nanotubes having different diameters [34]. It should also be noted that the nanotube diameter dependence is much weaker for the G’ band than for the RBM band [35].

**Figure 1:**
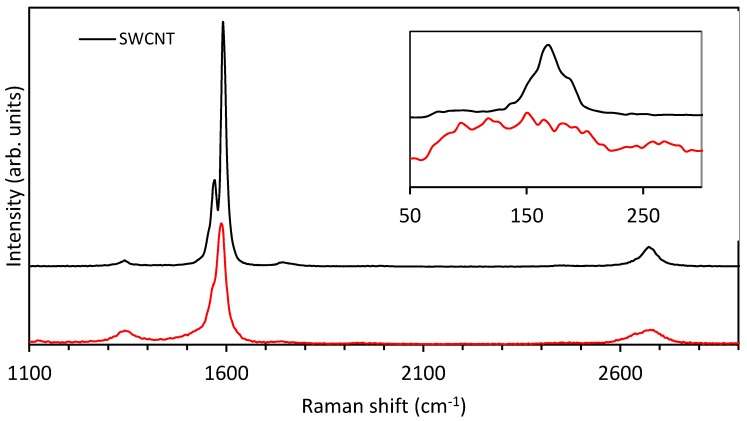
Raman spectra for a single-walled carbon nanotube (SWCNT) and a double-walled carbon nanotube (DWCNT) with inset showing the radial breathing mode (RBM) region.

Figure 1 (black line) is a single Raman spectrum of the SWCNT sample on the gold-chromium coated alumina substrate. The doublet within the G band is a characteristic of SWCNTs; the G^−^ peak at 1570 cm^−1^ and the G^+^ peak at 1590 cm^−1^ [31,36]. The G:D ratio is 48.14 ± 14.19 (or, as is also used, the D:G ratio = 0.02 ± 0.006) indicating the surface is dominated by sp^2^ hybridisation. Figure 1 (red line) is a single Raman spectrum of the DWCNT sample on a gold-chromium coated alumina substrate. The D band for these DWCNTs is broader than the SWCNT and the G:D ratio is 5.294 ± 5.177 (or D:G ratio = 0.189 ± 0.185) giving evidence for the presence of defects with sp^3^ hybridisation. The radial breathing mode (RBM) for the SWCNT has a sharp peak focussed around 170 cm^−1^ whilst the DWCNT RBM is very broad, ranging from 65 to 290 cm^−1^.

Given the position of the RBM peak is inversely proportional to the diameter of the CNTs (Equation (2)), it can be estimated from the RBM peak that the diameter of the SWCNT is approximately 1.38 nm (using prefactor A = 234 cm^−1^ nm and assuming B = 0, i.e., negligible environmental effect) [32]. The DWCNT sample displayed a broad range of RBM, with peaks ranging from 65 to 290 cm^−1^, indicating diameters from 0.8 to 2.6 nm. This is consistent with observations by Moore et al. [37] with the peaks in the region near, and including, the 65 cm^−1^ band corresponding to the outer walls (up to a maximum of 2.6 nm) and the peaks in the region near, and including, the 290 cm^−1^ band corresponding to the inner walls (down to a minimum of 0.8 nm). This suggests that the DWCNT sample has a larger diameter distribution, with more RBM constituent peaks [37].

ωRBM = A/dt + B,(2)

### 3.2. Analysis of Ultraviolet Photoelectron (UP) and Metastable Helium Induced Electron (MIE) Spectra

**Figure 2:**
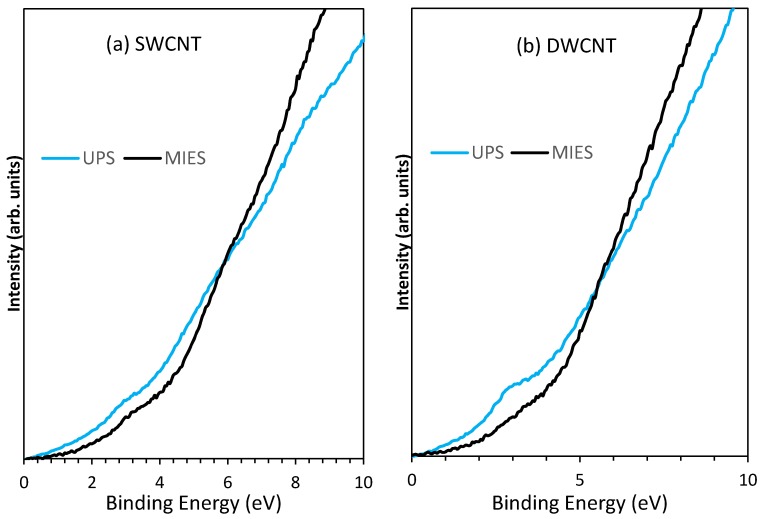
Comparison of ultraviolet photoelectron (UP) and metastable helium induced electron (MIE) spectra at low temperatures for both (**a**) SWCNT (113 K) and (**b**) DWCNT (173 K).

The UP and MIE spectra of SWCNTs (at 113 K) are presented in Figure 2a. Both the UP and the MIE spectra share the peak at 3 eV and a broad structure between 6 and 8 eV; the UPS has a peak evident at 8 eV. The 3 eV corresponds to the 2pπ states whilst the feature at between 6 and 8 eV relates to the 2pσ states [38,39].

The UP and MIE spectra for the DWCNTs (at 173 K) are presented in Figure 2b. The UP spectra, similar to the SWCNT, shows a feature at 3 eV corresponding to the 2pπ states [38,39] whilst the MIE spectra for DWCNTs seems to be depleted in the 3 eV region. The feature at 3 eV is specifically characteristic for the 2pπ states. It should be noted that the UP and MIE spectra for each of the SWCNTs and DWCNTs have been taken simultaneously, thus at the same temperature. The Raman results previously conducted on the DWCNTs indicated that the DWCNT sample had sp^3^ hybridisation and that the sp^2^ hybridisation is relatively low, thus the 2pπ states in the MIES would be low.

## 4. Discussion

UPS and MIES both examine the surface of the SWCNT and the DWCNT; however, due to the different interactions, MIES has a high surface sensitivity (only the outer most atoms) whilst UPS can penetrate a few nanometres, occasionally including the electron density of the substrate as well as the entire CNT cross-section. Reviewing the spectra in Figure 2b it could be stated that the MIE spectrum has indicated a limited population of 2pπ states on the outer layer of the DWCNT, supported by the Raman measurements, which indicated defects. The UP spectrum, however, still indicated a strong 2pπ signal similar to that seen in the SWCNT, which had very little defects. The UPS may be seeing the higher concentration of these states as it measures the inner tube within the DWCNT as well as the outside. It has previously been stated that the outer wall of a DWCNT is preferentially functionalised in a reaction leaving the inner wall DoS within the DWCNT intact; these measurements support this statement.

## 5. Conclusions

The UPS of DWCNTs provided information across the entire cross-section of the CNTs whilst the MIES provided information for the outer wall only. Combining the data from these two methods, the inner wall CNT valence electrons can be investigated, providing a better understanding of the internal electronic properties. This instrumental mechanism concluded that the DoS of the outer walls for the DWCNT was not favourable for use in electronic devices as it lost its ballistic conduction properties through possessing defects in the structure, however the inner wall of the DWCNT still maintained its population of low energy valence electrons desirable for ballistic conduction. This instrumental mechanism may also be considered for other materials composed of a few monolayers that experience different DoS at the outermost surface and immediately beneath the surface. The present work shows that combining surface analysis with MIES and UPS is specifically relevant for low-dimensional materials like CNTs and 2D materials.

## Supplementary Materials

The following are available online at http://www.mdpi.com/2079-4991/8/6/448/s1, Figure S1: Comparison of MIES at range of temperatures (Kelvin); as the temperature drops the features amplify, Figure S2: Variation of AD and AN/RI dominance across the temperature range (Kelvin); AD dominates at lower temperatures, Figure S3: C 1s XP spectra of the SWCNT and DWCNT. The C1 s spectra are similar to those published for HOPG and characteristic for sp^2^ hybridised C.
