# Supplementary material for: Measuring the Density of States of the Inner and Outer Wall of Double-Walled Carbon Nanotubes
Source: Nanomaterials (Basel). 2018 Jun 19;8(6):448. doi: 10.3390/nano8060448 (PMC6027179; doi:10.3390/nano8060448)
Supplement: Supplementary file 1 [file nanomaterials-08-00448-s001.pdf]

Supplementary Materials

Measuring the Density of States of the Inner and Outer Wall of Double Wall Carbon Nanotubes

Benjamin A. Chambers [1], Cameron Shearer [1,2], Leping Yu [1,] Christopher T. Gibson [1*] and Gunther G. Andersson [1*]

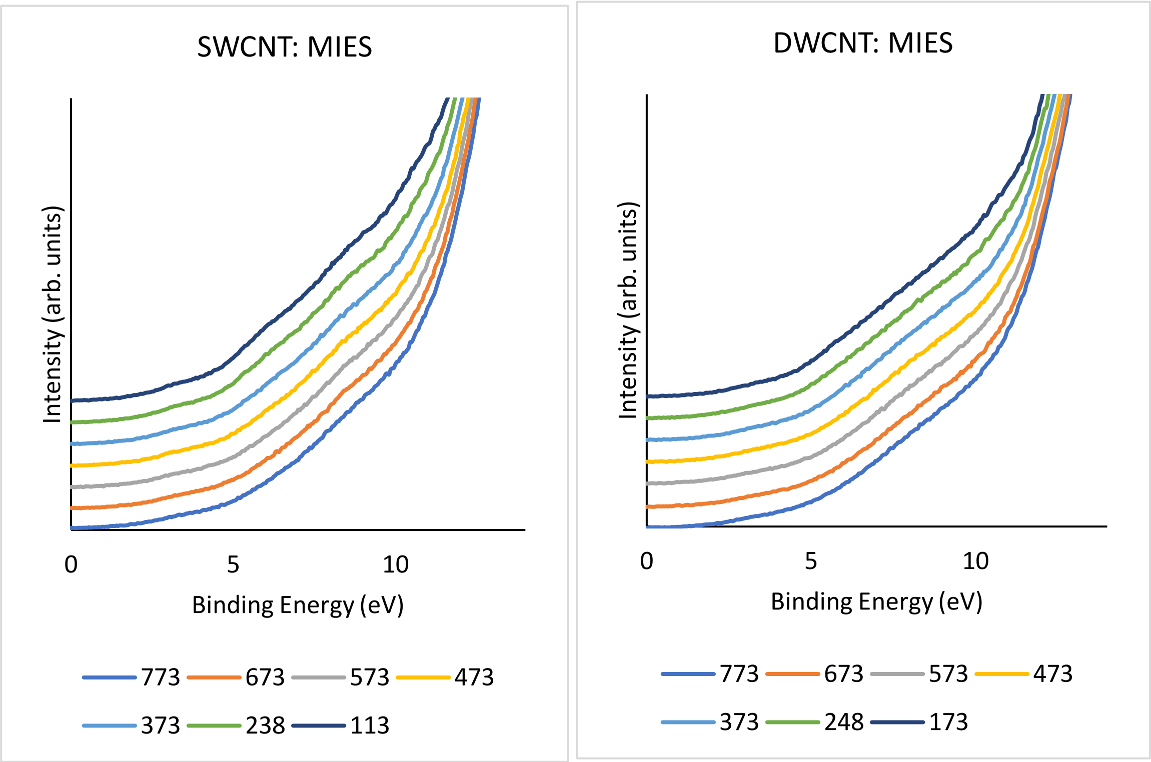

Figure S1. Comparison of MIES at range of temperatures (Kelvin); as the temperature drops the features amplify.

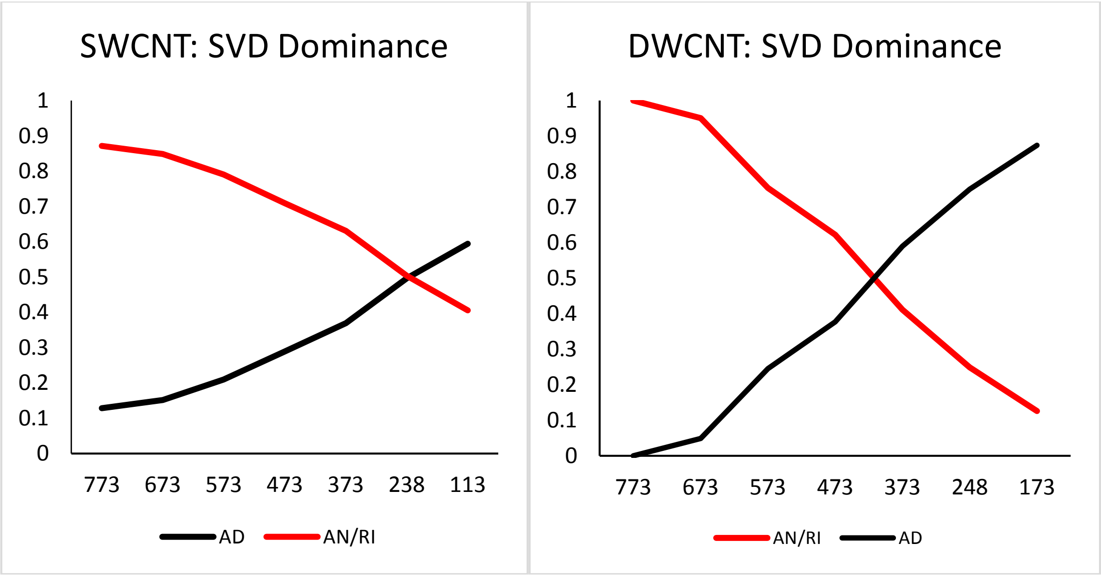

Figure S2. Variation of AD and AN/RI dominance across the temperature range (Kelvin); AD dominates at lower temperatures.

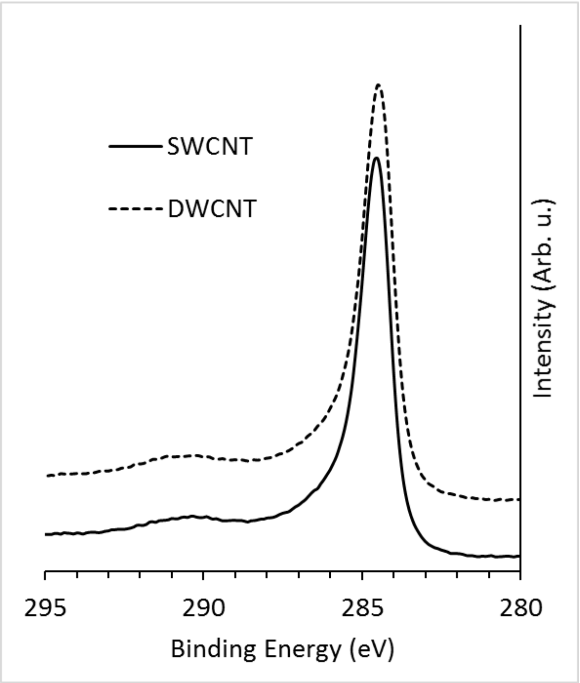

Figure S3. C 1s XP spectra of the SWCNT and DWCNT. The C 1s spectra are similar to those published for HOPG [1] and characteristic for sp^2 hybridised C. Specifically the position of the C 1s peak at 284.5 eV, the asymmetry to the higher binding energy and the broad feature around 291 eV are characteristic for sp^2 hybridisation.

References

1. Chambers, B.A., et al., The direct measurement of the electronic density of states of graphene using metastable induced electron spectroscopy. 2D Materials, 2017. 4(2): p. 025068.
